# Accuracy and Reproducibility of a Modified Echocardiographic Method for Right Ventricular Output Calculation in Neonates

**DOI:** 10.3390/jcdd12010018

**Published:** 2025-01-06

**Authors:** Carlotta Milocchi, Silvia Nogara, Giorgia Mazzuca, Federica Runfola, Martina Ciarcià, Iuri Corsini, Benjamim Ficial

**Affiliations:** 1 Neonatal Intensive Care Unit, Azienda Ospedaliera Universitaria Integrata Verona, 37126 Verona, Italy; carlotta.milocchi@gmail.com (C.M.); silvia.nogara92@gmail.com (S.N.); giorgiamazzuca@gmail.com (G.M.); federica.runfola@aovr.veneto.it (F.R.); martina.ciarci@gmail.com (M.C.); 2 Division of Neonatology, Careggi University Hospital of Florence, 50134 Florence, Italy; corsiniiuri@gmail.com

**Keywords:** right ventricular output, left ventricular output, systemic blood flow, neonate, newborn, neonatologist performed echocardiography, echocardiography, neonatal intensive care

## Abstract

We aimed to evaluate the accuracy and reproducibility of right ventricular output (RVO) using different anatomical landmarks: the internal pulmonary valve diameter (PVD) between the valve hinge points (hinge-PVD) according to the traditional technique, and PVD between the valve leaflet tips (tip-PVD). This was a retrospective analysis of prospective collected data. All neonates with echocardiographic measurements of RVO and left ventricular output (LVO) without congenital heart disease, including patent ductus arteriosus and patent foramen ovale > 3 mm, were included. Accuracy was assessed by comparison with LVO. Intra- and inter-observer reproducibility of the off-line analysis were assessed. Forty-five neonates were included. RVO calculation with tip-PVD was more accurate than hinge-PVD in comparison with LVO, r^2^ 0.712 versus 0.464, bias (95% limits of agreement) 1.4 mL/kg/min (−26–29 mL/kg/min) versus 61 mL/kg/min (−11–132 mL/kg/min), respectively. Both hinge-PVD and tip-PVD presented similar reproducibility, with an intra-observer bias (95% LOA) of 0.3 (−1.0–0.5) and –0.2 (−0.8–0.5) respectively, and an inter-observer bias of 0.1 (−1.3–1.6) and 0.1 (−1.4–1.6). RVO calculation using tip-PVD was more accurate than the conventional technique, with similar reproducibility.

## 1. Introduction

Shock is a state of impaired cellular energy synthesis when tissue oxygen delivery no longer satisfies demand. In the first phase of shock, perfusion and oxygen delivery are maintained towards the so-called vital organs (heart, brain, and adrenal glands) by selective regional vasodilation in combination with vasoconstriction to non-vital organs. This compensated stage of shock is the result of neuroendocrine mechanisms. As the product of cardiac output (which falls) and systemic vascular resistance (which increases), blood pressure actually remains in the normal range. When this redistribution fails, perfusion and oxygenation of the vital organs become impaired, resulting in systemic hypotension and multi-organ dysfunction. This is the phase of uncompensated shock [1].

To improve outcomes, it is mandatory to promptly identify neonates in compensated shock, where blood pressure is still in the normal ranges. Therefore, monitoring perfusion or cardiac output has become increasingly important [2].

Targeted Neonatal Echocardiography (TNE) or Neonatologist Performed Echocardiography (NPE), as it is called in Europe, is the most common method used at the bedside to measure cardiac output and to gain insights into the underlying pathophysiologic mechanisms of circulatory failure [1,3]. Left and right ventricular outputs (LVO and RVO) can be obtained to assess systemic and pulmonary blood flow [4]. However, these measurements should be interpreted with caution, as they are complicated by the presence of intra- and extracardiac shunts [5].

RVO has been extensively adopted in the routine clinical practice in neonatal intensive care units (NICU) as a measurement of systemic blood flow (SBF) [6,7]. In fact, in the absence of a significant shunt through the PFO, RVO represents the incoming blood from both the inferior and superior vena cava (SVC). Therefore, it is a valuable measure of SBF, even in neonates with a patent ductus arteriosus (PDA), in whom LVO, rather than assessing SBF, is a surrogate of pulmonary overcirculation.

However, the current guidelines on NPE do not recommend the use of RVO as a measure of SBF at the cot-side, because RVO has not been validated against magnetic resonance imaging (MRI) and has a huge variability between and within observers [3,8]. The main source of error is the measurement of pulmonary valve diameter (PVD) [9].

**Figure 1:**
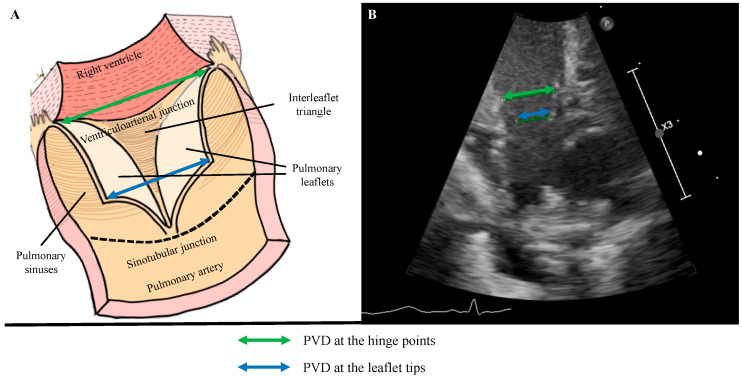
(**A**) Schematic illustration of the pulmonary root after 3D-reconstruction showing a parasternal long-axis view of the pulmonary valve from the ventriculoarterial junction to the sinotubular junction. Pulmonary leaflets, interleaflet triangles and sinuses are shown. (**B**) Echocardiographic parasternal long axis 2D view of the pulmonary valve. The internal diameter between the valve hinge points or hinge-PVD (green) and between the leaflet tips or tip-PVD (blue) are shown. Abbreviations: PVD = pulmonary valve diameter.

Conventionally, PVD is measured at the valve hinge points (hinge-PVD). However, we noticed that the leaflets usually have a conical rather than a cylindrical shape and the diameter between the leaflet tips (tip-PVD) is shorter compared to the hinge-PVD (Figure 1).

We hypothesized that tip-PVD may be more accurate compared to hinge-PVD, because it reflects the smaller orifice which determines the velocity of blood ejected by the right ventricle.

Our aim was to assess the accuracy of the RVO calculated from hinge-PVD (hinge-RVO) and tip-PVD (tip-RVO), using LVO as a comparison, in neonates without PDA. Secondly, we evaluated the intra- and inter-observer reproducibility.

## 2. Materials and Methods

We conducted a retrospective observational study at the level III academic NICU of Verona, from January 2023 to August 2024. Ethics approval was obtained from the Local Ethics Committee. Parents or guardians of all included infants provided written informed consent. The study was reported following the related guidelines [10].

We assessed prospectively recruited term and preterm healthy neonates for eligibility, in whom NPE was performed within the first month of life, outside the transitional period, where ductus arteriosus is often patent. Cardio-respiratory healthiness was defined as follows: no need for supplemental oxygen and/or respiratory support, except for babies with gestational age (GA) ≤ 32 weeks, in whom non-invasive respiratory support with a FiO_2_ < 0.3 was accepted, as previously reported [11,12,13].

Inclusion criteria were informed parental consent, cardio-respiratory healthiness and absence of hemodynamically significant PDA (hsPDA).

Exclusion criteria were suspected intrauterine infection, suspected sepsis, major congenital abnormalities, congenital heart disease (except for PFO), small or large for gestational age, intrauterine growth retardation, pulmonary hypertension, need for inotropes and/or vasopressors and image quality rated poor or unusable (see below).

Accuracy was assessed by comparing tip-RVO and hinge-RVO with LVO. Intra- and inter-observer reproducibility of the off-line analysis were assessed in a random sample of neonates. Hinge and tip-PVD measurements were repeated by the primary observer (C.M.) 4 weeks after the first measurements and by a second observer (B.F.) blinded to previous data, as reported [11,14].

### 2.1. Clinical Data

The following clinical data were collected at the baseline: GA, birth weight, sex, type of delivery, 5 min Apgar score, prenatal corticosteroids, umbilical cord pH, and base excess. Characteristics of respiratory support, if any, and hydration pro kg were collected.

### 2.2. Echocardiography

All echocardiographic examinations were performed using a Philips Epiq 7 system with a 12 MHz probe (Philips Ultrasound, Andover, MA, USA). Images were acquired according to the recommendations of the American Society of Echocardiography [8]. Electrocardiograms were recorded simultaneously, and three cardiac cycles were digitally stored using a high frame rate (100–130 Hz). Image quality was independently assessed according to Colan et al. [15]. Images rated poor or unusable were excluded.

The analysis of digitally stored images was performed offline using Tomtec Arena, version TTA2 41.00 (Tomtec, Unterschleißheim, Germany). The echocardiographic measurements were carried out offline by an observer (C.M.) blinded to the clinical data: RVO, LVO.

LVO was calculated as previously described [1,8,16]. Firstly, the aortic valve was imaged with high-definition zoom from the parasternal long-axis view and the internal diameter at the valve hinge points was measured at end-systole. Secondly, aortic flow velocity was assessed by pulsed-wave Doppler from an optimized apical five-chamber view, with the pulsed-wave Doppler gate placed at the level of the aortic valve. Care was taken to minimize angulation between the Doppler beam and flow direction, to obtain an angle of insonation as close to zero as possible and definitely <20° [8].

The RVO was calculated as follows: pulmonary flow velocity was assessed by pulsed-wave Doppler from a modified parasternal long-axis view (tilted toward the left shoulder), with the pulsed-wave Doppler gate placed at the level of the pulmonary valve (PV). Care was taken to obtain an angle of insonation as close to zero as possible and definitely <20°. Then, the PVD was measured at end-systole at two different anatomical landmarks: the internal diameter between the valve hinge points (hinge-PVD) and the internal diameter between the tips of the valve leaflets (tip-PVD) (Figure 1).

### 2.3. Statistical Analysis

Data were summarized as the mean and standard deviation (SD) for variables with a symmetrical distribution, and as the median [Q1, Q3 range] for variables exhibiting a skewed distribution. Categorical variables were summarized as frequencies (percentages). Linear regression analysis and Bland–Altman analysis were used to assess accuracy of the conventional and modified technique for RVO assessment. The sample size for the reproducibility assessment was calculated considering a minimum acceptable reliability of 0.7, number of raters per subject of 2 and assuming an expected reliability of 0.9. To reach an 80% power at 0.05 level, the sample size was 23 scans [17]. Intra- and inter-observer reproducibility were assessed using: (a) Bland–Altman analysis (mean bias and limits of agreement, LOA); (b) intraclass correlation coefficient (ICC) with 95% confidence intervals (CI); (c) coefficient of variation (COV). COV (%) was calculated as follows: (standard deviation of differences between repeated measurements/arithmetic mean of all repeated measurements) × 100%. *p* values < 0.05 were considered significant. Data were analyzed with SPSS 20 (SPSS, Chicago, IL, USA).

## 3. Results

**Figure 2:**
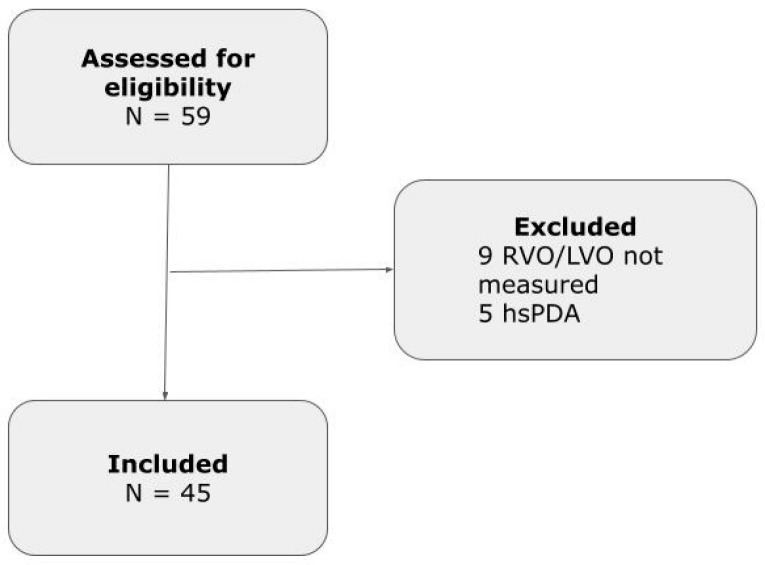
Study Flowchart.

We assessed 59 neonates for eligibility. Fourteen neonates did not meet the inclusion criteria: nine neonates were excluded, since digitally stored clips to assess RVO and/or LVO were not retrieved. Five infants were also excluded, being diagnosed with hsPDA (Figure 2). Finally, 45 infants with a mean ± SD gestational age of 35.4 ± 3.8 weeks and a birth weight of 2380 ± 933 g were included.

### 3.1. Demographic Data

Clinical characteristics of enrolled neonates are detailed in Table 1.

Echocardiographic recordings were rated excellent, good or fair following Colan classification. Specifically, PV clips were rated excellent (18%), good (38%), fair (44%); pulmonary VTI were rated excellent (24%), good (62%), fair (13%). None of the echocardiographic clips was excluded and/or received a score of 4–5 (poor or unusable quality).

Table 2 shows clinical data at the time of echocardiography, including respiratory support, blood pressure, and hydration. None of the infants enrolled was hemodynamically unstable.

### 3.2. Echocardiographic Data

Table 3 shows the echocardiographic measurements of the infants at the time of echocardiography.

### 3.3. Accuracy of Tip-RVO and Hinge-RVO

**Figure 3:**
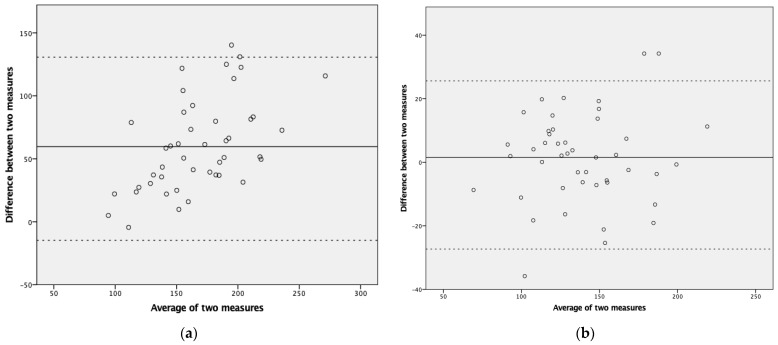
Bland–Altmann Plot of RVO (**a**) with pulmonary valve diameter measured at the valve hinge points (hinge-RVO) and LVO; (**b**) with pulmonary valve diameter measured at the leaflet tips (tip-RVO) and LVO.

The accuracy of hinge-RVO and tip-RVO was assessed using LVO as a comparison. Bland–Altman, COV, and linear regression analysis were performed. When comparing hinge-RVO to LVO, the Bland–Altman analysis showed a bias of 61 mL/kg/min, LOA ranging from −11 to 132 mL/kg/min and a COV of 22.9% (Figure 3a). When comparing tip-RVO to LVO, Bland–Altman analysis showed a bias of 1.4 mL/kg/min, LOA ranging from −26 to 29 mL/kg/min and a COV of 9.6% (Figure 3b).

**Figure 4:**
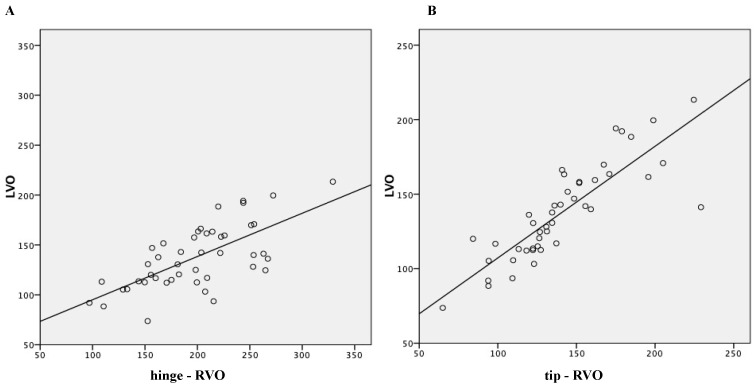
Linear regression analysis: (**A**) RVO with pulmonary valve diameter measured at the valve hinge points (hinge-RVO) and LVO, r^2^ = 0.464; (**B**) RVO with pulmonary valve diameter measured at the leaflet tips (tip-RVO) and LVO, r^2^ = 0.715.

Linear regression analysis results are shown in Figure 4A,B: tip-RVO was more accurate than hinge-RVO, in comparison with LVO, with r^2^ of 0.712 versus 0.464 respectively.

We presented the comparison between the aortic diameter measured at the hinge points and at the leaflet tips, similarly to PVD, as a Supplementary Materials. The difference between the two diameters, contrary to PVD, is clinically irrelevant.

### 3.4. Intra- and Inter-Observer Reproducibility

Reproducibility analysis of hinge-PVD and tip-PVD are shown in Table 4. Both diameters showed similar variability. Intra-observer bias (95% LOA) was 0.3 (−1.0–0.5) for hinge-PVD and –0.2 (−0.8–0.5) for tip-PVD; inter-observer bias was –0.3 (−1.8–1.2) for hinge-PVD and 0.1 (−1.4–1.6) for tip-PVD.

## 4. Discussion

This study aims to evaluate the accuracy and reproducibility of a modified echocardiographic approach to measure RVO, compared to the conventional method.

RVO has gained increased attention from clinicians, especially neonatologists, due to its capability to assess SBF, even in patients with PDA. In fact, an accurate assessment of systemic perfusion is crucial in preterm infants, in whom PDA is common. RVO represents the incoming blood from both the inferior and superior vena cava (SVC), thus offering a valuable measure of SBF. The current guidelines discourage the use of RVO as a measure of SBF at the cot-side, because RVO has not been validated and has a huge variability between and within observers [1].

The modified approach arises from clinical observations of the motion of PV during systole, when the leaflets present a conical shape instead of a cylindrical one. The tip-PVD represents the narrowest diameter of the right ventricular outflow tract, which is critical for determining the cross-sectional area through which blood flows. Therefore, we hypothesized that tip-PVD may be a more accurate measurement of PV diameter to calculate RVO.

Previous studies have explored the structure and anatomy of PV [18,19,20]. PV has substantial differences with aortic valve in both anatomy and function (Figure 1). First, PV has a less defined and thinner fibrous structure compared to the aortic valve. Second, contrary to the aortic valve, PV is entirely anchored to the myocardium, which is thinner and provides a more delicate attachment [19,20]. Third, the sinotubular junction, which delimits cranially the pulmonary sinuses from the pulmonary artery, only has a subtle line and not a well-defined circular ring of dense connective tissue, like the aortic root. Lis et al. found that, at the level of the sinotubular junction, there is the smallest possible area of PV, they called tubular plane [21]. Although there is paucity of data in adults and no data in neonates, we believe that these subtle differences play an important role in the opening and closing dynamics of PV and may explain our findings. Further morphological studies are needed to detail the anatomy of PV in neonates.

The most significant finding of this study is the superior accuracy of the modified method or tip-RVO compared to the conventional approach. In stable neonates, with no significant shunts such as PDA, RVO should be equal to LVO.

The mean difference or bias between tip-RVO and LVO was negligible compared to hinge-RVO, with tighter LOA. This result underscores the limitations of using the conventional method to measure RVO as a measure of SBF and suggests a promising alternative technique, that provides a more accurate estimation of RVO and, consequently, SBF.

As previously noted [9], the measurement accuracy in newborns is seldom reported. Studies that do address measurement accuracy often face limitations, including small sample sizes, a limited number of observers for comparison, and inconsistent reporting of reliability and agreement metrics. Moving forward, it is essential to enhance research efforts aimed at assessing the accuracy of neonatal measurements to validate existing data.

Moreover, this study reported the intra-observer and inter-observer variability in measuring RVO diameter for both the conventional and modified methods, confirming previous findings. Hinge and tip-PVD presented similar variability. We confirm previous findings of Alfarano et al. [7], who recently demonstrated a significant inter-observer variability in PVD measurements using the conventional method (bias—0.38, LOA from −1.36 to 0.60) and in RVO measurements (22.1 mL/kg/min, LOA from −104 to 59.8 mL/kg/min). Inter-observer variability for RVO has also been reported in newborn infants by Tsai-Goodman et al. and Popat. Tsai-Goodman et al. [22] found a mean difference (bias) of 0.3 mL/kg/min between observers for RVO, (LOA from −24.1 to 23.4 mL/kg/min). Popat reported a mean difference of −26.6 mL/kg/min (LOA −131.4 to 78.2) [9].

As de Boode et al. and Alfarano et al. observed [1,7], the disappointing accuracy and repeatability of RVO measurements is probably related to the difficulty in the exact measurement of the cross-sectional area (CSA), which is derived from measuring PVD to obtain the radius, which is subsequently squared; the assumption of a perfect circular form of the outflow tract; the inaccuracy in the assessment of the VTI; the assumption of laminar blood flow; and the error secondary to the angle of insonation.

Many studies [22,23] have previously reported differences in LVO and RVO measurements in infants without any significant cardiac shunts. On the whole, they concluded that this was likely related to inaccuracies in the measurement of the diameters rather than differences in angle of insonation. Given these premises, it is crucial to improve the accuracy of RVO measurement in neonates and we believe that incorporating tip-PVD in the calculation of RVO could represent a significant step forward.

This study has several limitations. First, the sample size was relatively small and from a single center. The latter may have led to selection bias, impairing the generalizability of study findings. Nevertheless, similar previous studies on validation of echocardiographic measures of blood flow in neonates had similar sample sizes [7,11,22,24,25,26,27]. Further studies are needed to confirm our findings and to establish reference ranges.

Secondly, we included stable neonates in the study, and that may differ from critically ill neonates in whom RVO is measured for clinical purposes and that may have overall worse image quality. These factors may limit the generalizability of study findings. The internal validation of RVO assumed that RVO equals LVO. The latter is true only in stable neonates; thus, the cardiorespiratory stability was a necessary inclusion criterion. We acknowledge that this is a potential limitation of the study. However, we feel that the combination of validation against LVO together with intra- and inter-observer repeatability still provides data of interest to support the adoption of tip-PVD. Further studies are needed to confirm our findings.

Thirdly, we did not assess scan–rescan echo repeatability. However, a number of other studies on neonatal echocardiography presented reproducibility data only from off-line analyses [11,14].

Fourthly, we acknowledge that the echocardiographic LVO assessment is not the optimal gold standard. However, from a pragmatical point of view, it is the only comparator available in this fragile category of patients and a number of studies used ultrasound LVO measurements to validate other techniques of cardiac output monitoring, such as bioimpedance, bioreactance, transcutaneous Doppler, etc. [11,28,29,30].

Finally, we did not consider the potential role of the angle of insonation on the assessment of pulmonary blood flow velocity and RVO calculation. However, PVD has been shown to be the most important source of variability [9]. Future studies are needed to address this point.

## 5. Conclusions

This study highlights the significant advantages of the modified method for measuring RVO over the conventional approach, demonstrating improved accuracy and similar variability. The findings underscore the limitations of the conventional method in clinical practice, where precise assessment of SBF is essential.

The data suggest that incorporating tip-PVD into the RVO calculation provides a more reliable estimation of SBF, which could enhance clinical decision-making at the cot-side, especially in patients with conditions such as PDA. However, further studies are needed to confirm our findings. To improve the reliability of RVO, it is crucial to conduct more extensive studies with larger sample sizes and standardized measurement protocols. Such efforts will help confirm the findings of this study and support the adoption of a validated method for RVO measurement in neonatal care.

In summary, while RVO has the potential to be a valuable tool in assessing systemic perfusion, particularly in neonates with PDA, further studies are needed to confirm its validity and reliability in clinical settings.

## Figures and Tables

**Table 1:** Demographic and clinical data at the baseline.

Clinical Characteristics	
Gestational age (week)	35.4 ± 3.8
Birth weight (g)	2380 ± 933
Sex (F)	23 (51.1%)
Cesarean section	32 (71.1%)
5 APGAR score	9 [2–10]
Antenatal steroids	12 (26.6%)

Values are presented as mean ± SD, median [range] and count (%).

**Table 2:** Clinical data at the time of echocardiography.

Clinical Data at the Time of Echocardiography	
Current weight (g)	2306 ± 877
Postnatal age (hours)	24 [2–168]
Fluids (mL/kg/die)	110 ± 45
Systolic blood pressure (mmHg)	71 ± 8
Mean blood pressure (mmHg)	50 ± 8
Diastolic blood pressure (mmHg)	41 ± 8
Respiratory support:	
None	35 (77.7%)
CPAP	4 (8.8%)
Non-invasive ventilation	7 (15.5%)
FiO_2_ (%)	21 [21–28]

Values are presented as mean ± SD, median [range] and count (%). Abbreviations: CPAP = continuous positive airway pressure; FiO_2_ = fraction of inspired oxygen).

**Table 3:** Echocardiographic measurements of LVO and RVO.

**Left Ventricular Output (LVO)**	
Aortic VTI (cm)	10.4 ± 2.8
Aortic HR (bpm)	135 ± 17
Aortic diameter (mm)	5.5 ± 0.8
LVO (mL/kg/min)	138 ± 32
**Right Ventricular Output (RVO)**	
Pulmonary Valve VTI (cm)	11.2 ± 2.8
Pulmonary HR (bpm)	134 ± 20
tip-PVD (mm)	5.1 ± 0.9
tip-RVO (mL/kg/min)	141 ± 36
hinge-PVD (mm)	6.1 ± 1.0
hinge-RVO (mL/kg/min)	198 ± 50

Values are presented as mean ± SD, median [range] and count (%). Abbreviations: HR = heart rate; PVD = pulmonary valve diameter, VTI = velocity time integral.

**Table 4:** Reproducibility analysis of pulmonary valve diameter measured at the hinge points and at the leaflet tips in a cohort of neonates.

	Intra-Observer Reproducibility			Inter-Observer Reproducibility		
	Bias (95% LOA)	ICC (95% CI)	COV (%)	Bias (95% LOA)	ICC (95% CI)	COV (%)
Pulmonary valve diameter at the hinge points (mm)	−0.3 (−1.0 to 0.5)	0.94 (0.88 to 0.98)	6	−0.3 (−1.8 to 1.2)	0.73 (0.35 to 0.88)	12
Pulmonary valve diameter at the tips (mm)	−0.2 (−0.8 to 0.5)	0.94 (0.86 to 0.97)	6	0.1 (−1.4 to 1.6)	0.70 (0.30 to 0.87)	14.3

All ICC *p* values < 0.01. Abbreviations: CI = confidence intervals; COV = coefficient of variation, ICC = intra-class correlation coefficient; LOA = limits of agreement.

## Data Availability

Data are available upon reasonable request.

## Supplementary Materials

The following supporting information can be downloaded at: https://www.mdpi.com/article/10.3390/jcdd12010018/s1, Table S1: Comparison of aortic valve diameter at the hinge points and at the leaflet tips; Table S2: Bland-Altman analysis of aortic valve diameter measured at the hinge points and at the leaflet tips.

## References

[1] de Boode W.P., van der Lee R., Eriksen B.H., Nestaas E., Dempsey E., Singh Y., Austin T., El-Khuffash A. (2018). The role of Neonatologist Performed Echocardiography in the assessment and management of neonatal shock. Pediatr. Res..

[2] Schwarz C.E., Dempsey E.M. (2020). Management of Neonatal Hypotension and Shock. Semin. Fetal Neonatal Med..

[3] Groves A.M., Singh Y., Dempsey E., Molnar Z., Austin T., El-Khuffash A., de Boode W.P. (2018). Introduction to neonatologist-performed echocardiography. Pediatr. Res..

[4] El-Khuffash A.F., McNamara P.J. (2011). Neonatologist-performed functional echocardiography in the neonatal intensive care unit. Semin. Fetal Neonatal Med..

[5] Barrington K., El-Khuffash A., Dempsey E. (2020). Intervention and Outcome for Neonatal Hypotension. Clin. Perinatol..

[6] Corsini I., Ficial B., Fiocchi S., Schena F., Capolupo I., Cerbo R.M., Condò M., Doni D., La Placa S., Porzio S. (2019). Neonatologist performed echocardiography (NPE) in Italian neonatal intensive care units: A national survey. Ital. J. Pediatr..

[7] Alfarano A., Marzollo R., Bosio M.I., Tomasi C., Codega A., Picciau L., Motta M., Risso F.M. (2024). Inter-observer variability of right ventricular output measurement in newborn infants: An observational study. Int. J. Cardiovasc. Imaging.

[8] McNamara P.J., Jain A., El-Khuffash A., Giesinger R., Weisz D., Freud L., Levy P.T., Bhombal S., de Boode W., Leone T. (2024). Guidelines and Recommendations for Targeted Neonatal Echocardiography and Cardiac Point-of-Care Ultrasound in the Neonatal Intensive Care Unit: An Update from the American Society of Echocardiography. J. Am. Soc. Echocardiogr..

[9] Popat H., Robledo K.P., Sebastian L., Evans N., Gill A., Kluckow M., Sinhal S., de Waal K., Tarnow-Mordi W., Osborn D. (2018). Interobserver agreement and image quality of functional cardiac ultrasound measures used in a randomised trial of delayed cord clamping in preterm infants. Arch. Dis. Child.-Fetal Neonatal Ed..

[10] Vandenbroucke J.P., Von Elm E., Altman D.G., Gøtzsche P.C., Mulrow C.D., Pocock S.J., Poole C., Schlesselman J.J., Egger M. (2007). Strengthening the Reporting of Observational Studies in Epidemiology (STROBE): Explanation and elaboration. Epidemiology.

[11] Ficial B., Bonafiglia E., Gangemi A., Clemente M., Cappelleri A., Corsini I., Biban P. (2022). Impact of Aortic Diameter Measurements at Three Anatomical Landmarks on Left Ventricular Output Calculation in Neonates. J. Ultrasound Med..

[12] Koestenberger M., Nagel B., Ravekes W., Urlesberger B., Raith W., Avian A., Halb V., Cvirn G., Fritsch P., Gamillscheg A. (2011). Systolic Right Ventricular Function in Preterm and Term Neonates: Reference Values of the Tricuspid Annular Plane Systolic Excursion (TAPSE) in 258 Patients and Calculation of Z-Score Values. Neonatology.

[13] Koestenberger M., Nagel B., Ravekes W., Gamillscheg A., Pichler G., Avian A., Heinzl B., Binder C., Cvirn G., Urlesberger B. (2013). Right Ventricular Performance in Preterm and Term Neonates: Reference Values of the Tricuspid Annular Peak Systolic Velocity Measured by Tissue Doppler Imaging. Neonatology.

[14] Ficial B., Corsini I., Clemente M., Cappelleri A., Remaschi G., Quer L., Urbani G., Sandrini C., Biban P., Dani C. (2022). Feasibility, Reproducibility and Reference Ranges of Left Atrial Strain in Preterm and Term Neonates in the First 48 h of Life. Diagnostics.

[15] Colan S.D., Shirali G., Margossian R., Gallagher D., Altmann K., Canter C., Chen S., Golding F., Radojewski E., Camitta M. (2012). The Ventricular Volume Variability Study of the Pediatric Heart Network: Study Design and Impact of Beat Averaging and Variable Type on the Reproducibility of Echocardiographic Measurements in Children with Chronic Dilated Cardiomyopathy. J. Am. Soc. Echocardiogr..

[16] Ficial B., Finnemore A.E., Cox D.J., Broadhouse K.M., Price A.N., Durighel G., Ekitzidou G., Hajnal J.V., Edwards A.D., Groves A.M. (2013). Validation study of the accuracy of echocardiographic measurements of systemic blood flow volume in newborn infants. J. Am. Soc. Echocardiogr..

[17] Walter S.D., Eliasziw M., Donner A. (1998). Sample size and optimal designs for reliability studies. Stat. Med..

[18] Stamm C., Anderson R.H., Yen S.H., Yen Ho S. (1998). Clinical Anatomy of the Normal Pulmonary Root Compared With That in Isolated Pulmonary Valvular Stenosis. J. Am. Coll. Cardiol..

[19] Hokken R.B., Bartelings M.M., Bogers A.J.J.C., Gittonberger-De Groot A.C. (1997). Morphology of the pulmonary and aortic roots with regard to the pulmonary autograft procedure. J. Thorac. Cardiovasc. Surg..

[20] Sievers H.H., Hemmer W., Beyersdorf F., Moritz A., Moosdorf R., Lichtenberg A., Misfeld M., Charitos E.I. (2012). The everyday used nomenclature of the aortic root components: The tower of babel?. Eur. J. Cardio-Thorac. Surg..

[21] Lis M., Krawczyk-Ożóg A., Hołda J., Tyrak K., Dudkiewicz D., Yakovliev A., Strona M., Bolechała F., Jakiel R., Jakiel M. (2023). Pulmonary valve morphometry revisited: Clinical implications for valvular and supravalvular interventions. Clin. Anat..

[22] Tsai-Goodman B., Martin R.P., Marlow N., Skinner J.R. (2001). The repeatability of echocardiographic determination of right ventricular output in the newborn. Cardiol. Young.

[23] de Boode W.P., Singh Y., Molnar Z., Schubert U., Savoia M., Sehgal A., Levy P., McNamara P., El-Khuffash A. (2018). Application of Neonatologist Performed Echocardiography in the assessment and management of persistent pulmonary hypertension of the newborn. Pediatr. Res..

[24] Beker F., Davis P.G., Sehgal A., Rogerson S. (2014). Echocardiographic assessment of left ventricular outflow tract diameter in preterm infants. Australas. J. Ultrasound Med..

[25] Sloot S.C., De Waal K.A., Van Der Lee J.H., Van Kaam A.H. (2010). Central blood flow measurements in stable preterm infants after the transitional period. Arch. Dis. Child. Fetal Neonatal Ed..

[26] Patel N., Dodsworth M., Mills J.F. (2011). Cardiac output measurement in newborn infants using the ultrasonic cardiac output monitor: An assessment of agreement with conventional echocardiography, repeatability and new user experience. Arch. Dis. Child. Fetal Neonatal Ed..

[27] Ficial B., Bonafiglia E., Padovani E.M., Prioli M.A., Finnemore A.E., Cox D.J., Broadhouse K.M., Price A.N., Durighel G., Groves A.M. (2017). A modified echocardiographic approach improves reliability of superior vena caval flow quantification. Arch. Dis. Child. Fetal Neonatal Ed..

[28] Cappelleri A., Bussmann N., Harvey S., Levy P.T., Franklin O., El-Khuffash A. (2020). Myocardial function in late preterm infants during the transitional period: Comprehensive appraisal with deformation mechanics and non-invasive cardiac output monitoring. Cardiol. Young.

[29] Fraga M.V., Dysart K.C., Rintoul N., Chaudhary A.S., Ratcliffe S.J., Fedec A., Kren S., Cohen M.S., Kirpalani H. (2019). Cardiac Output Measurement Using the Ultrasonic Cardiac Output Monitor: A Validation Study in Newborn Infants. Neonatology.

[30] Van Wyk L., Gupta S., Lawrenson J., de Boode W.P. (2022). Accuracy and Trending Ability of Electrical Biosensing Technology for Non-invasive Cardiac Output Monitoring in Neonates: A Systematic Qualitative Review. Front. Pediatr..

