# Supplementary material for: Accuracy and Reproducibility of a Modified Echocardiographic Method for Right Ventricular Output Calculation in Neonates
Source: J Cardiovasc Dev Dis. 2025 Jan 6;12(1):18. doi: 10.3390/jcdd12010018 (PMC11765522; doi:10.3390/jcdd12010018)
Supplement: Supplementary file 1 [file jcdd-12-00018-s001.zip › jcdd-3372937-supplementary.pdf]

Supplemental Table 1
Comparison of aortic valve diameter at the hinge points and at the leaflet tips.

	Aortic valve diameter at the hinge points (mm)	Aortic valve diameter at the tips (mm)	p
**Mean ± DS**	5.13 ± 0.69	5.15 ± 0.71	0.521

Supplemental Table 2

Bland-Altman analysis of aortic valve diameter measured at the hinge points and at the leaflet tips

	Bias (mm)	95% LOA (mm)	RI (%)	r^2
	- 0.02	-0.3 to 0.2	6	0.93
